# Reply to “Catch rate composition affects assessment of protected area impacts”

**DOI:** 10.1038/s41467-021-21608-3

**Published:** 2021-03-11

**Authors:** John Lynham, Anton Nikolaev, Jennifer Raynor, Thaís Vilela, Juan Carlos Villaseñor-Derbez

**Affiliations:** 1 Department of Economics, University of Hawai’i at Mānoa, Honolulu, HI USA; 2 Information and Computer Sciences, University of Hawai’i at Mānoa, Honolulu, HI USA; 3 Department of Economics, Wesleyan University, Middletown, CT USA; 4 Conservation Strategy Fund, Washington, DC USA; 5 Bren School of Environmental Science and Management, University of California, Santa Barbara, CA USA

**Keywords:** Conservation biology, Environmental economics

**Replying to** J. R. Sweeney. *Nature Communications* 10.1038/s41467-021-21607-4 (2021)

We thank Dr. Sweeney for taking the time to confirm the results of our analysis. Our work evaluated the economic impact of the expansion of two Marine National Monuments on the Hawai’i deep-set longline fishing fleet, which targets tuna species^1^. We used catch of bigeye and yellowfin tuna per unit of effort as our primary outcome variable. We focused on bigeye and yellowfin tuna since these are the target species and (as confirmed by Dr. Sweeney’s Fig. 1b) constitute 80–90% of the revenue in this fishery. While yellowfin tuna ranks fourth in terms of numbers caught between 2010 and 2017, it is second in terms of pounds landed and revenue generated. Bigeye tuna ranks first for both pounds landed and revenue.

It is certainly interesting to consider whether there have been impacts on other, less commercially important, species. But catch-per-unit-effort for 11 different species combined is misleading as an “accurate assessment of economic impacts” since it treats each species as equally valuable. This is definitely not true: for example, in 2017 the value of one bigeye tuna was about 18 times that of an escolar. Catching 5000 fewer bigeye tuna is not offset by catching 5000 more escolar.

Therefore, any “robust measure” that combines multiple species should weight those species by their economic value (in other words, revenue-per-unit-effort). This is the analysis shown in panel b of Dr. Sweeney’s Fig. 1. The horizontal blue and yellow lines show that revenue-per-unit-effort has generally increased since the expansions began in 2014 and the blue-dashed and yellow-dashed lines show that revenue-per-unit-effort further increased for the 16 months following the Papahānaumokuākea monument expansion (the time period that our study focused on). This directly contradicts Dr. Chan’s study^2^ which, looking at the exact same 16-month period, claimed that the Papahānaumokuākea monument expansion caused a 9% decrease in revenue-per-unit-effort.

Dr. Sweeney recommends a reanalysis of our approach using his more robust outcome measure (revenue-per-unit-effort). This is a good idea because correctly attributing the increase in revenue-per-unit-effort following both expansions is complicated by environmental, climate, and other drivers of change that may have occurred at the same time as the expansions. We present the results of the suggested reanalysis in Table 1 that includes controls for environmental and other drivers of change (this is essentially a replication of Table 2 from our original paper^1^). Our conclusions are unchanged: we do not observe any evidence of negative impacts from either monument expansion. In fact, combined with the new evidence in Dr. Sweeney’s Figure 1b, it now appears that the Papahānaumokuākea expansion actually benefited the Hawai’i tuna fishery (Column (6)). This is a new and exciting finding. Dr. Sweeney is correct: catch rate composition affects assessment of protected area impacts. It makes the conclusions of our assessment even stronger and undermines claims that there have been negative impacts from the Papahānaumokuākea expansion. In particular, we would caution against attributing the slight drop in revenue-per-unit-effort that occurs five years after the expansion of the Pacific Remote Islands monument and three years after the expansion of the Papahānaumokuākea monument to either monument expansion, without carefully controlling for all of the external factors that could have changed over this longer time horizon.

**Table 1:** Difference-in-differences estimation.

	(1)	(2)	(3)	(4)	(5)	(6)
PRI Expansion	234.168^***^	−213.234^**^	−342.525^***^			
	(79.873)	(95.598)	(100.419)			
PMNM Expansion				−757.274^***^	−824.478^***^	−873.576^***^
				(150.793)	(158.847)	(198.830)
Hawaii-based Tuna Trips	−3390.484^***^	−3235.953^***^	−3231.547^***^	−4033.577^***^	−4041.807^***^	−2473.943^***^
	(40.092)	(40.365)	(53.138)	(63.730)	(63.037)	(229.100)
PRI * Hawaii	−0.033	−13.160	13.300			
	(83.224)	(79.379)	(85.988)			
PMNM * Hawaii				997.133^***^	979.787^***^	732.643^***^
				(152.867)	(153.312)	(173.837)
Month Dummies	No	Yes	Yes	No	Yes	Yes
Year Dummies	No	Yes	Yes	No	Yes	Yes
Vessel Dummies	No	No	Yes	No	No	Yes
Additional Controls	No	No	Yes	No	No	Yes
Observations	33,444	33,444	33,444	34,964	34,964	34,964
*R*^2^	0.313	0.346	0.382	0.273	0.284	0.329

This table is a replication of Table 2 in our original paper^1^. The only change is the dependent variable used. The new dependent variable in all columns is Revenue per 1000 Hooks. Revenue is calculated by multiplying each fish by its average weight and price per pound for that year. Unlike Dr. Sweeney, we only have access to data on 7 species categories, not the full 11 mentioned in his comment. These are bigeye tuna, yellowfin tuna, swordfish, skipjack tuna, albacore tuna, bluefin tuna, and unidentified tuna. PRI stands for the Pacific Remote Islands Marine National Monument and PMNM stands for the Papahānaumokuākea Marine National Monument. In Columns (1)–(3), the control group is Hawaii-based swordfish trips and the sample runs from January 1st 2010 to August 25th 2016. In Columns (4)–(6), the control group is American Samoa-based tuna trips and the sample runs from January 1st 2010 to December 31st 2017. The Additional Controls are whether the set included an experimental component, a dummy variable for whether the WCPFC waters were closed to fishing, a dummy variable for whether IATTC waters were closed to vessels longer than 24 m, Monthly El Nino indicator, Monthly El Nino indicator lagged by 1 year, Monthly El Nino indicator lagged by 2 years, and Monthly El Nino indicator lagged by 3 years. Please refer to the Methods section of the original paper for more details. ^*^p < 0.1; ^**^p < 0.05; ^***^p < 0.01 for two-sided *t*-test of statistical significance using heteroskedasticity-robust standard errors (which are presented in parentheses).

## Data Availability

The Observer Program data used in Table 1 are available from NOAA Fisheries https://inport.nmfs.noaa.gov/inport/item/21854 but restrictions apply to the availability of these data, which contain business confidential information. Under the terms of a non-disclosure agreement with NOAA, J.L. can not make these data publicly available.

## Code availability

All code used to generate the table in this study are publicly available on GitHub at the following url: https://github.com/lynham/monuments.
